# Loneliness as a predictor of self-rated health: a gendered, cross-national analysis in six European countries

**DOI:** 10.3389/fsoc.2026.1726387

**Published:** 2026-04-21

**Authors:** Ana G. Padrón Armas, Josué Gutiérrez-Barroso, Esther Torrado Martín-Palomino, Rafael Serrano del Rosal

**Affiliations:** 1 Instituto de Estudios Sociales Avanzados, Consejo Superior de Investigaciones Científicas (IESA-CSIC), Córdoba, Spain; 2 Departamento de Sociología y Antropología, Universidad de La Laguna, San Cristóbal de La Laguna, Spain

**Keywords:** gender inequalities, loneliness, public health, self-rated health (SRH), sociodemographic factors

## Abstract

**Background:**

Loneliness is a recognized social determinant of health with substantial physical and psychological implications. Self-rated health (SRH) is a widely used population indicator, with strong predictive validity for morbidity and mortality.

**Objective:**

To examine whether loneliness is associated with SRH across six European countries (Germany, Greece, Ireland, Poland, Spain and Sweden), incorporating a gender perspective and the moderating role of sociodemographic factors.

**Methods:**

Using a quota-based, cross-sectional sample (N = 4,800), we conducted descriptive analyses by country, sex, and age group, and estimated nested OLS regression models (treating SRH as a 1–5 scale) to examine the association between SRH and loneliness.

**Results:**

Higher loneliness was consistently associated with poorer SRH across all models, even after adjusting for sociodemographic and contextual covariates. Women reported lower SRH and higher loneliness, most pronounced at younger ages, while socioeconomic status emerged as the strongest structural correlate of SRH.

**Conclusion:**

Loneliness is robustly associated with poorer SRH and exhibits cross-national variation, yet gender gaps remain salient. Findings underline the need for targeted public health interventions addressing loneliness, with particular emphasis on gender equity and protection for the most vulnerable groups.

## 1 Introduction

Self-rated health (SRH) is a core indicator for monitoring population health and inequalities, as highlighted by World Health Organization (2010a) and Organisation for Economic Co-operation and Development (OECD) (2021). The value of SRH is twofold: first, it consistently predicts morbidity, functional decline and mortality, even after adjustment for clinical and sociodemographic covariates (Mossey and Shapiro, 1982; Idler and Benyamini, 1997; Vie et al., 2019; Lorem et al., 2020); second, it captures psychosocial and cultural dimensions relevant to the subjective experience of health (DeSalvo et al., 2006; Jylhä, 2009). In parallel, loneliness has emerged as a first-order social determinant of health, associated to poorer physical and mental outcomes, especially salient in urbanized, ageing societies, and with serious health risks, comparable in magnitude to other deeply studied classic behavioral factors (Hawkley and Cacioppo, 2010; Holt-Lunstad et al., 2015; Hajek et al., 2023). Accordingly, the WHO Commission on Social Connection has identified loneliness as a priority challenge because of its association with cardiovascular disease, diabetes, cognitive decline and premature mortality across the life course (World Health Organization, 2025). In Europe, the European Commission Loneliness Survey has also documented the rising prevalence of loneliness and its marked heterogeneity by age, gender and sociocultural context (European Commission, 2022).

It is crucial to distinguish social isolation, an objective state measurable via frequency of contacts and participation, from loneliness, which is a subjective experience (European Commission, Joint Research Centre, 2023). Strictly defined, unwanted loneliness reflects a discrepancy between desired and actual social relationships (Peplau and Perlman, 1982). The notion of “social loneliness” has also been proposed to capture sparse or unsatisfactory networks (Yanguas et al., 2018) and, among older adults, isolation–loneliness associations have been modelled contextually (Wenger et al., 1996). This article focuses on loneliness as a perceived, subjective feeling.

Gender inequalities in health intensify when SRH is considered: across Europe, women report worse health despite their longer life expectancy (Gorman and Read, 2006; Morcillo Cebolla et al., 2014), a pattern known as the “female morbidity paradox” (Verbrugge, 1989; Macintyre et al., 1996). This pattern aligns with gendered social constructs: men tend to under-express distress and avoid help-seeking, in line with male ideals of self-sufficiency and toughness (Connell and Messerschmidt, 2005; Springer and Mouzon, 2011), whereas women, who are more susceptible to stereotypes of fragility, report poorer SRH and greater recognition of psychological distress (Ruiz and Verbrugge, 1997; Yokopenic et al., 1983; Bacigalupe and Martín, 2021; Torrado Martín-Palomino, 2022; Bacigalupe et al., 2020). Loneliness is also gendered: women more often report emotional loneliness, whereas men more often experience structural isolation, with cultural variations (Barreto et al., 2021). Response styles (acquiescence and extreme response) may further shape gender differences in SRH (Hibbing et al., 2017; Weijters et al., 2010) with stigma constraining open acknowledgement of loneliness (Yanguas et al., 2018). Consequently, the WHO recommends treating gender as a structural determinant rather than as a mere demographic covariate, given its influence on resource access, risk exposure and health trajectories (World Health Organization, 2010b).

Loneliness is often described as following a “U-shape” according to age (higher among the younger and the elderly) and further conditioned by socioeconomic status and emotional state, as in depression (Luhmann and Hawkley, 2016; Yanguas et al., 2018; Victor and Yang, 2012). Recent European evidence corroborates age-related gradients in loneliness (Pagán, 2020; Pagán et al., 2024). In Europe, older women face a higher risk of persistent loneliness (Boehlen et al., 2023; Hajek et al., 2023) though they often maintain broader networks (Pinquart and Sorensen, 2001). The COVID-19 pandemic intensified isolation and distress, especially among the youth, with marked increases in loneliness and affective symptoms (Ahrendt et al., 2021; Kuric et al., 2020; Marchini et al., 2021; Christ and Gray, 2022; Espinoza and Hernandez, 2022; Vidal Fernández and Halty, 2020). Cohabitation is not uniformly protective, living with others did not always shield individuals from malaise (“cohabitation paradox”) (Simón et al., 2021). In contrast, religiosity may buffer loneliness by providing social ties and a sense of meaning, with benefits for well-being (Lee and Newberg, 2005). Finally, socioeconomic status is consistently associated with both loneliness and SRH, reinforcing the social gradient in health (Marmot, 2005; Niedzwiedz et al., 2016a).

Over time, research on SRH has moved beyond biomedical factors to incorporate nonclinical predictors that shape how individuals appraise their health. One of the most outstanding contributions to this new field of study is Kang’s (2023), which traces how the values of personality traits evolve and reveal themselves in SRH across adulthood. Following their lead, this study examines loneliness, a non-clinical, socially embedded determinant, in relation to SRH. This article tests whether loneliness is associated with SRH in six European countries (Germany, Spain, Greece, Ireland, Poland, and Sweden), incorporating a gender perspective and adjusting for structural covariates. The primary hypothesis is that greater loneliness is associated with poorer SRH, and that this association persists after controlling for sociodemographic factors. We additionally hypothesize that women will report poorer SRH and greater loneliness than men.

Overall, the article contributes recent comparative gender-aware evidence, while integrating multiple social determinants and speaking to the “social dimension of health” (del Serrano Rosal, 2024). The findings offer operational bases for designing public health strategies that are sensitive to gender and the life course, while tailored to regional specificities.

## 2 Data and methods

### 2.1 Study design and population

We conducted an observational, cross-sectional, comparative study in six European countries (Germany, Spain, Greece, Ireland, Poland, and Sweden). The design, using harmonized validated instruments, enabled us to estimate the association between loneliness and SRH and to compare patterns across sociodemographic subgroups and national contexts.

The target population comprised adults aged 18 years and older residing in the six countries. Countries were purposively selected to maximize heterogeneity along structural dimensions relevant to health and the social dimension of pain (e.g., gender regimes, health-care systems, welfare state models, and socioeconomic–cultural context, including income inequality).

Sampling relied on a quota-based design implemented through professionally managed online panels. Fieldwork was conducted from 22 May to 17 June 2025, yielding 4,800 completed self-administered online interviews (800 per country). Quotas were set by sex, age, and education, including crossed quotas (sex×age; age×education) to approximate each country’s population structure. Standard quality controls were applied by the fieldwork provider; 414 completed questionnaires were removed for failing quality criteria prior to delivery of the final dataset. Eligibility criteria were age ≥18, residence in one of the six countries, and provision of informed consent.

Ethical approval was granted by the Institutional Ethics Committee of the Spanish National Research Council (CSIC; Ref. 271/2023). All participants provided informed consent prior to the survey. Analyses used anonymized data exclusively for research purposes.

### 2.2 Measures

#### 2.2.1 Self-rated health (SRH)

SRH was assessed by one single standard item, “In general, would you say your health is…?,” with five response options (Very bad, Bad, Fair, Good, Very good) coded from 1–5 with higher scores indicating better SRH. This one-item measure is widely accepted, given its strong predictive validity for morbidity, functional decline and mortality, plus its capacity to capture the psychosocial and cultural dimensions of perceived health (Ware and Sherbourne, 1992; Cullati et al., 2020). Following common practice in comparative population research, supported by evidence of reliability (Lundberg and Manderbacka, 1996), SRH was modelled as a continuous variable (1–5) in OLS regressions.

#### 2.2.2 Loneliness (UCLA-3)

Loneliness was measured with the Three-Item UCLA Loneliness Scale (UCLA-3), the shorter version of the original 20-item scale (Russell, 1996) designed for large surveys due to its brevity, low respondent burden and robust cross-national psychometrics (Hughes et al., 2004). The items were “I lack companionship,” “I feel left out,” and “I feel isolated,” with responses 1 = hardly ever/never, 2 = some of the time, 3 = often. Items were summed to yield a total score from 3 to 9 (higher = greater loneliness). In line with standard practice [Hughes et al., 2004; Steptoe et al., 2013; Office for National Statistics (ONS), 2018; National Academies of Sciences, Engineering, and Medicine (NASEM), 2020], we analyzed the total score and referred to the measure as Loneliness (UCLA-3).

Unidimensionality was assessed first with Exploratory Factor Analysis (EFA) using Unweighted Least Squares (ULS/minres) without rotation, followed by Confirmatory Factor Analysis (CFA) for ordinal indicators estimated with Diagonally Weighted Least Squares (DWLS) (Rosseel, 2012). Because with three indicators a single-factor CFA is just-identified (df = 0), global fit indices are not informative and are reported only for completeness; our use of CFA is limited to obtaining standardized loadings and residuals, while evidence for unidimensionality relies primarily on the EFA and internal consistency metrics. Following conventional guidelines (Hu and Bentler, 1999), we report the Comparative Fit Index (CFI), Tucker–Lewis Index (TLI), Root Mean Square Error of Approximation (RMSEA; 90% CI when available), and the Standardized Root Mean Square Residual (SRMR). Given the three-item specification, fit indices may cluster at boundary values (e.g., CFI/TLI ≈ 1.00; RMSEA ≈ 0.00) and should be interpreted cautiously, as recommended for very short scales (Bandalos, 2018). Reliability was examined using Cronbach’s alpha, McDonald’s omega (Cronbach, 1951; McDonald, 1999), and ordinal alpha computed from the polychoric correlation matrix (Zumbo et al., 2007; Gadermann et al., 2012; Cronbach, 1951; McDonald, 1999). Internal consistency was further assessed via corrected items–total correlations: each item correlated with the sum of the remaining two items (Field, 2013; Tavakol and Dennick, 2011).

### 2.3 Covariates

We included standard sociodemographic covariates, either the originals used in the questionnaires (sex; country; economic situation on a five-point ordinal scale) or recoded to enhance interpretability and cross-country comparability: age by decade groups; education as a three-level ordinal variable; and employment status, religiosity and cohabitation as dichotomous indicators. Countries were entered as dummy variables, with Sweden as the reference category (criterion: lowest SRH observed). The exact categories and references are specified in the results tables.

### 2.4 Statistical analysis

Analyses were conducted with IBM SPSS Statistics (v27) and jamovi (The Jamovi Project, 2024; v2.6): two-sided *α* = 0.05, with significance denoted as *p < 0.05, **p < 0.01, ***p < 0.001. Fit indices were rounded to two decimals (RMSEA to two decimals and, when available, the 90% CI). Interpretive thresholds are provided for orientation (Hu and Bentler, 1999) but were not used as rigid cutoffs.

First, we produced descriptive statistics (means, SDs, proportions) and compared means across country, sex, and age groups. Then, we proceeded to estimate three nested OLS models, with SRH (1–5) as the dependent variable: Model 1 (baseline) included Loneliness (UCLA-3) as the focal predictor (model-based statistical term; non-causal in this cross-sectional design); Model 2 (adjusted) added the questionnaire values for sex, country and economic situation; and Model 3 (full) additionally included age, education, employment status, religiosity and cohabitation. We reported unstandardized *B* and standardized *β* coefficients, 95% confidence intervals when relevant, *R*^2^ and adjusted *R*^2^, and overall *F* and *p*-statistics.

Regression models were estimated using complete-case analysis within each specification (listwise deletion). Therefore, due to item nonresponse on the study variables and covariates, the analytic sample size varies across nested models as additional covariates are introduced. All analyses were conducted using the post-stratification weight (ponde).

As a robustness check for the ordinal nature of SRH, we re-estimated the fully adjusted specification using ordinal regression (ordered logit) with the same covariate set; results are reported in Supplementary Table S1.

## 3 Results

Table 1 summarizes the psychometric performance of the UCLA-3. Exploratory factor analysis supported a unidimensional structure with homogeneous loadings. Because with three indicators a single-factor CFA is just-identified (df = 0), global fit indices are not informative; the near-boundary values (CFI/TLI ≈ 1.00; RMSEA ≈ 0.00; SRMR ≈ 0.00) are expected for very short scales (Bandalos, 2018) and are reported only for completeness. We use the CFA to report standardized loadings, whereas inferences about unidimensionality rest primarily on the EFA and reliability evidence. Reliability was strong across indices, which together justifies using the total score (3–9) as a valid summary measure for comparative analyses.

**Table 1:** Psychometric results for loneliness (UCLA-3).

Panel A. Items: descriptives, item total, and factor loadings				
Item	M (SD)	r_it	EFA loading (ULS)	CFA loading (β)
1. I lack companionship	1.86 (0.75)	0.546	0.635	0.721
2. I feel left out	1.66 (0.73)	0.897	0.805	0.878
3. I feel isolated	1.72 (0.74)	0.892	0.883	0.943

Panel B. Global scale indices		
Index	Value
Cronbach’s α	0.814–0.815	
Ordinal α	0.882	
McDonald ω	0.822–0.823	
AVE (Average Variance Extracted)	0.727	
Explained Variance (EFA)	61.00%	
KMO	0.684	
Bartlett *χ*^2^(df)	4,853 (3), *p* < 0.001	
CFA fit (DWLS)	CFI = 1.00; TLI = 1.00; RMSEA = 0.00; SRMR = 0.00	

Valid *n* (UCLA-3) = 4,330. Items scored 1–3; total = 3–9 (higher = greater loneliness). r_it denotes the corrected item–total correlation (each item with the sum of the other two). EFA: ULS/minres, no rotation. CFA: DWLS for ordinal indicators. Reliability: Cronbach’s α, McDonald’s ω, ordinal *α* (polychoric). Fit indices: CFI, TLI, RMSEA, SRMR. Given the three-item specification, near-boundary fit values (CFI/TLI ≈ 1.00; RMSEA ≈ 0.00) are expected and should be interpreted cautiously (Hu and Bentler, 1999; Bandalos, 2018). AVE, average variance extracted; KMO, Kaiser–Meyer–Olkin.

Table 2 reports the distribution by country of sociodemographic characteristics and study variables. Given the balanced sample by sex, this distribution does not differ significantly across countries. All other sociodemographic variables (age, education, economic situation, employment status, religiosity and cohabitation) differ significantly across countries. Cross-country differences are also observed for SRH (*p* < 0.001) and for the UCLA-3 items (*p* = 0.004).

**Table 2:** Sociodemographic characteristics and SRH/loneliness measures by country.

Variables	Germany (*n* = 799)	Greece (*n* = 798)	Ireland (*n* = 800)	Poland (*n* = 799)	Spain (*n* = 800)	Sweden (*n* = 800)	*p*-value
Sex							0.961
Female	0.51	0.52	0.51	0.52	0.52	0.50	
Male	0.49	0.49	0.49	0.48	0.49	0.50	
Age							<0.001
18–29	0.17	0.14	0.20	0.16	0.15	0.19	
30–39	0.16	0.13	0.18	0.17	0.17	0.18	
40–49	0.15	0.19	0.18	0.18	0.18	0.12	
50–59	0.15	0.23	0.14	0.16	0.19	0.14	
60–69	0.19	0.15	0.15	0.18	0.16	0.16	
70+	0.24	0.07	0.14	0.14	0.11	0.31	
Education level							0.000
No education or basic	0.19	0.24	0.15	0.09	0.40	0.15	
Secondary	0.51	0.47	0.37	0.60	0.25	0.42	
University	0.31	0.29	0.48	0.32	0.36	0.43	
Economic situation							0.000
Very good	0.08	0.04	0.08	0.03	0.05	0.08	
Good	0.35	0.21	0.32	0.33	0.39	0.28	
Fair	0.34	0.48	0.39	0.47	0.40	0.35	
Bad	0.15	0.19	0.15	0.13	0.12	0.16	
Very bad	0.08	0.08	0.06	0.04	0.04	0.13	
Employment status							0.001
Active	0.66	0.71	0.66	0.69	0.72	0.63	
Inactive	0.34	0.29	0.34	0.31	0.29	0.37	
Religiosity							0.000
Non-religious	0.39	0.15	0.27	0.17	0.34	0.61	
Religious	0.62	0.85	0.73	0.83	0.66	0.40	
Cohabitation							<0.001
Lives alone	0.30	0.13	0.12	0.18	0.15	0.38	
Lives with others	0.70	0.87	0.88	0.82	0.85	0.62	
SRH							<0.001
Very good	0.09	0.16	0.14	0.06	0.13	0.09	
Good	0.37	0.36	0.47	0.36	0.39	0.29	
Fair	0.37	0.34	0.31	0.37	0.34	0.36	
Bad	0.16	0.11	0.06	0.18	0.11	0.20	
Very bad	0.02	0.02	0.03	0.03	0.02	0.05	
UCLA-3							0.004
I lack companionship	0.14	0.28	0.12	0.11	0.14	0.12	
I feel left out	0.10	0.10	0.11	0.10	0.13	0.15	
I feel isolated	0.14	0.11	0.16	0.12	0.13	0.13	

Proportions. *p*-values correspond to ANOVA or chi-square tests, as appropriate to the variable type. *n* = 4,800.

Table 3 reported mean scores (standard deviations) for SRH and loneliness by country and sex, together with the corresponding sex differences. For SRH, women scored slightly worse than men in every country; the largest gaps occurred in Spain (−0.22) and Sweden (−0.23). For loneliness, patterns are more heterogeneous: women reported greater loneliness in all countries except Poland, where men showed a slightly higher mean (−0.15). The largest differences are observed in Germany (+0.29) and Ireland (+0.23), followed by Spain (+0.19), while gaps are minimal in Sweden and Greece.

**Table 3:** Mean (SD) of SRH and Loneliness by country and sex.

Variable	Total/sex/dif	Germany	Greece	Ireland	Poland	Spain	Sweden
SRH	Total	3.37 (0.97)	3.57 (0.98)	3.64 (0.89)	3.27 (0.95)	3.52 (0.96)	3.19 (1.03)
	Female	3.37 (0.99)	3.50 (0.97)	3.60 (0.91)	3.25 (0.94)	3.41 (0.94)	3.08 (1.08)
	Male	3.38 (0.95)	3.64 (1.00)	3.70 (0.86)	3.29 (0.96)	3.63 (0.98)	3.31 (0.98)
	F-M	−0.01	−0.14	−0.10	−0.04	−0.22	−0.23
Loneliness	Total	5.07 (1.91)	5.56 (1.64)	5.57 (1.91)	5.14 (1.92)	4.87 (1.95)	5.20 (1.92)
	Female	5.21 (1.96)	5.58 (1.56)	5.69 (1.88)	5.06 (1.89)	4.96 (1.91)	5.22 (1.92)
	Male	4.92 (1.84)	5.53 (1.72)	5.46 (1.93)	5.21 (1.95)	4.77 (2.00)	5.18 (1.92)
	F-M	0.29	0.05	0.23	−0.15	0.19	0.04

SRH scale: 1 = worst to 5 = best (higher scores indicate better self-rated health); Loneliness (UCLA-3) scale: 3 = not lonely to 9 = very lonely (higher scores indicate greater loneliness); Sex differences: computed as mean for women minus mean for men.

Table 4 displays SRH and loneliness by age group and sex. SRH was seen to decline progressively with age. Loneliness, conversely, revealed the opposite pattern, with higher levels at younger ages and a gradual decade-by-decade decline. Women reported poorer SRH than men across all age groups; differences were small but consistent. For loneliness, women scored higher at every age; the gap was less marked in youth and midlife (*Δ* = +0.02 to +0.21) and wider at ages 70 + (Δ = +0.79).

**Table 4:** Mean (SD) of SRH and loneliness by age group and sex.

Age group	Sex	SRH	Δ (F–M)	Loneliness	Δ (F–M)
18–29	Female	3.49 (0.93)	−0.18	6.04 (1.68)	0.11
	Male	3.67 (0.89)		5.94 (1.81)	
30–39	Female	3.46 (0.96)	−0.26	5.81 (1.83)	0.21
	Male	3.72 (0.81)		5.61 (1.92)	
40–49	Female	3.38 (0.93)	−0.13	5.57 (1.83)	0.02
	Male	3.51 (0.96)		5.55 (1.95)	
50–59	Female	3.23 (0.95)	−0.10	5.09 (1.84)	0.11
	Male	3.33 (1.01)		4.98 (1.87)	
60–69	Female	3.28 (1.01)	0.02	4.58 (1.78)	0.06
	Male	3.26 (0.95)		4.53 (1.66)	
70+	Female	3.28 (0.90)	0.02	4.61 (1.78)	0.79
	Male	3.26 (0.82)		3.82 (1.33)	
Total	Female	3.35 (0.96)	−0.12	5.29 (1.87)	0.11
	Male	3.47 (0.94)		5.18 (1.91)	

Total *N* = 4,758 (SRH) and 4,328 (Loneliness). Scales: SRH 1–5 (higher = better self-rated health); Loneliness 3–9 (higher = greater perceived loneliness). Δ SRH/Δ Loneliness = women–men mean difference (positive = women > men; negative = women < men).

Loneliness was seen to be a negative and statistically significant correlate of SRH across all specifications (Table 5). The association remained robust after adjustment for sociodemographic and structural covariates (fully adjusted: *β* = −0.224, *p* < 0.001). Age was shown to bear a graded, inverse relation with SRH: from age 40 onward, perceived health declined progressively, with the steepest deficits observed at ages 50–59 and 60–69. Sex differences were small but consistent, with men reporting slightly better SRH than women, even after full adjustment (*β* = 0.039, *p* < 0.01). Cross-national contrasts indicated higher SRH in Ireland and Greece relative to Sweden (reference), with Germany and Spain also showing smaller but significant advantages. Structural resources accounted for meaningful inter-individual differences. The economic situation exhibited the largest positive association (very good economy: *β* = 0.321, *p* < 0.001). Being employed, reporting no religiosity and having tertiary education associated positively with SRH, whereas cohabitation showed a slight negative association. In ordinal regression (ordered logit), loneliness remained associated with SRH (*b* = −0.255, SE = 0.018, *p* < 0.001; OR = 0.78 per 1-point increase in UCLA-3), supporting the robustness of the main OLS findings (Supplementary Table S1).

**Table 5:** OLS regression models of SRH on loneliness and sociodemographic variables.

Variable (ref.)	Model 1 B (β)	Model 2 B (β)	Model 3 B (β)
Loneliness	−0.117 (−0.235)***	−0.155 (−0.311)***	−0.112 (−0.224)***
Sex (ref = Female)	–	0.092 (0.049)**	0.074 (0.039)**
Age (ref = 18–29)	–		
30–39	–	−0.026 (−0.010)	−0.080 (−0.032)
40–49	–	−0.235 (−0.096)***	−0.266 (−0.108)***
50–59	–	−0.460 (−0.210)***	−0.385 (−0.176)***
60–69	–	−0.515 (−0.211)***	−0.383 (−0.158)***
70+	–	−0.505 (−0.143)***	−0.383 (−0.106)***
Country (ref = Sweden)			
Poland	–	0.056 (0.022)	−0.021 (−0.008)
Germany	–	0.175 (0.069)**	0.133 (0.052)**
Spain	–	0.295 (0.117)***	0.242 (0.099)***
Greece	–	0.454 (0.179)***	0.443 (0.175)***
Ireland	–	0.487 (0.194)***	0.399 (0.162)***
Education (ref = None/basic)	–	–	
Secondary	–	–	0.046 (0.024)
University	–	–	0.162 (0.084)***
Economic situation (ref = Very bad)	–	–	
Bad	–	–	0.379 (0.141)***
Fair	–	–	0.655 (0.341)***
Good	–	–	0.853 (0.426)***
Very good	–	–	1.261 (0.321)***
Employment status (ref = Inactive)	–	–	0.210 (0.103)***
Religiosity (ref = Yes)	–	–	0.160 (0.079)***
Cohabitation (ref = No)	–	–	−0.082 (−0.034)*
Constant	4.035***	4.234***	3.089***
*R* ^2^	0.055	0.142	0.249
Adjusted *R*^2^	0.055	0.140	0.245
*N*	4,304	4,302	3,844

Dependent variable SRH (1 = very bad, 5 = very good). Standardized coefficients (β) in parentheses. Reference category for each variable shown in parentheses.

## 4 Discussion

Psychometric results for the UCLA-3 indicated adequate internal consistency and support for a unidimensional structure, consistent with prior validation studies (Hughes et al., 2004). Given the brevity of scale, we interpret near-perfect CFA fit indices cautiously, as short scales can inflate fit statistics (Bandalos, 2018).

Our findings underline the salience of loneliness as a social determinant of health. Consistent with the reviewed literature, greater loneliness is associated with poorer SRH, even after adjusting for sex, age, and structural factors, consistent with prior evidence that frames loneliness as a relevant social risk marker for health (Holt-Lunstad et al., 2015). In accordance, the regression models show statistically significant negative associations between loneliness and SRH; this coincides with EU evidence (European Commission, 2022, EU-LS) which similarly documents a strong co-occurrence between loneliness and poor SRH.

Cross-country patterns are heterogeneous. Contrary to a simple South/East gradient as suggested by the EU-LS 2022 (European Commission, 2022), our six-country comparison yielded a more variegated profile. Notably, Ireland’s high levels of loneliness in our data mirror the EU-LS 2022, which also highlighted Greece. This distribution suggests that, beyond broad regional patterns, institutional arrangements (e.g., welfare regimes) and cultural norms may shape loneliness and SRH (Esping-Andersen, 1990; Bambra, 2007). Possible pathways include differences in social protection, opportunities for social participation, and health-care accessibility, as well as cultural norms that may shape both loneliness and SRH appraisal; given the small number of countries, we present these as interpretive hypotheses rather than tested macro-level effects.

With respect to the age gradient, SRH declines with advancing age, while loneliness is higher among youth and decreases with age. Rather than the commonly cited U-shape (Luhmann and Hawkley, 2016; Yanguas et al., 2018; Victor and Yang, 2012), the present pattern aligns more closely with the “youth loneliness paradox” (Qualter et al., 2015), where life transitions, labor market instability and social precarity heighten vulnerability in early adulthood. Given the post-pandemic timing of our data collection, elevated loneliness among younger adults may plausibly reflect lingering social and economic disruptions, although this study cannot test this mechanism directly. While causal inferences are not warranted, these patterns reinforce calls for youth-focused connection strategies in the aftermath of COVID-19. These results invite broadening the policy focus beyond older adults to include younger groups.

Gender inequalities are systematic, with women reporting poorer SRH and greater loneliness (Pinquart and Sorensen, 2001; Gorman and Read, 2006; Morcillo Cebolla et al., 2014; Yang and Victor, 2011). A social constructionist lens helps to explain this pattern: unequal care burdens, precarious employment and dense, yet demanding social networks may elevate loneliness and adversely affect health appraisals among women (Connell and Messerschmidt, 2005; Springer and Mouzon, 2011; Ruiz and Verbrugge, 1997; Bacigalupe and Martín, 2021). Moreover, the gender gap in loneliness widens at older ages in our data, nuancing narratives that assign greater structural loneliness to men (Barreto et al., 2021).

When structural resources are considered, the association between loneliness and SRH attenuates but remains significant, while the economic situation emerges as the strongest correlate of SRH. This is consistent with the social gradient in health (Marmot, 2005) and suggests that part of the loneliness–SRH associations may operate through pathways shared with socioeconomic disadvantage, such as constrained participation opportunities, material hardship and chronic stress, coherent with evidence that lower socioeconomic position relates to greater loneliness and poorer health (Niedzwiedz et al., 2016b). Employment and education are positively associated with SRH, consistent with mechanisms of social integration, self-sufficiency and information resources (Zajacova et al., 2017). By contrast, cohabitation shows a slightly negative association, which may reflect heterogeneity in relationship quality and household stressors not captured by cohabitation indicator (Simón, 2021). Non-religiosity was positively associated with SRH.

### 4.1 Limitations

This study has several limitations that should be considered when interpreting the findings. First, the design is cross-sectional, so temporal ordering cannot be established and the association between loneliness and SRH may be bidirectional. Accordingly, results should be interpreted as associations rather than causal effects. Second, an online panel was used for fieldwork. However, to safeguard data quality: (1) a panel with ISO 26362 certification, specifically for online access panels, was used, guaranteeing a quality standard for managing research panels; and (2) sample subjects were selected using stratified sampling by country, with uniform allocation across six European countries, and within each country, cross-quotas for sex, age, and educational level proportional to the population of each country. Despite this, we are aware that the panel’s recruitment may underrepresent individuals with limited digital access and those who are more socially isolated, with potential implications for the generalizability and magnitude of the observed associations. Third, analytic sample sizes decreased across nested models due to item nonresponse and complete-case estimation, which could introduce selection bias if missingness is systematic. Finally, the survey did not include a validated measure of mental health, which may confound and/or mediate the loneliness–SRH association; residual confounding therefore cannot be ruled out.

## 5 Conclusion

This comparative study indicates that loneliness is robustly associated with poorer SRH, net of observed covariates. The association persists after adjustment for sex, age and national context, consistent with loneliness as a salient social determinant of perceived well-being. Among the structural factors of influence, economic status is most closely associated with SRH, suggesting shared pathways connecting material disadvantage, opportunities for social participation and health, consistent with the partial attenuation of the loneliness effect when socioeconomic variables are considered.

Patterns are uneven across groups: younger adults exhibit higher levels of loneliness, and women consistently report worse SRH and greater loneliness than men, with magnitudes varying by country. Moving beyond the conventional framing of loneliness as primarily an issue of old age, youth emerge as a public health concern, while persistent gender gaps warrant attention.

Taken together, the findings point to the role of cultural norms, gendered social roles and the distribution of resources in shaping both loneliness and its health implications. Policy responses should prioritize young adults, women, and socioeconomically disadvantaged groups, consistent with the observed patterns in loneliness and self-rated health (SRH). It is particularly important to integrate brief loneliness identification tools not only in primary care and public health services, but also in educational settings and social/community services, alongside clear referral pathways to community participation and social integration resources. A life-course and gender-sensitive approach is needed to avoid “one-size-fits-all” solutions that risk perpetuating inequalities.

## Data Availability

The datasets analyzed in this article were collected within the project “El dolor en su contexto social” (PID2022-137976NB-I00). De-identified data will be made publicly available in an open repository after the project’s dissemination phase. Until then, data are available from the corresponding author on reasonable request.
