# Supplementary material for: Loneliness as a predictor of self-rated health: a gendered, cross-national analysis in six European countries
Source: Front Sociol. 2026 Apr 21;11:1726387. doi: 10.3389/fsoc.2026.1726387 (PMC13138951; doi:10.3389/fsoc.2026.1726387)
Supplement: Supplementary file 1 [file Data_Sheet_1.pdf]

Supplementary Table S1. Ordinal regression robustness check (ordered logit).

Predictor (ref)	b	SE	Wald	p	OR	95% CI OR
Loneliness	-0.255	0.018	198.987	<.001	0.775	0.748–0.803
Sex (ref = Female)	0.154	0.061	6.322	0.012	1.166	1.035–1.314
Age (ref = 18–29)						
30–39	-0.194	0.111	3.054	0.081	0.824	0.662–1.024
40–49	-0.586	0.11	28.338	<.001	0.557	0.448–0.691
50–59	-0.863	0.105	68.032	<.001	0.422	0.344–0.518
60–69	-0.873	0.113	59.378	<.001	0.418	0.335–0.522
70+	-0.904	0.152	35.293	<.001	0.405	0.300–0.546
Country (ref = Sweden)						
Poland	-0.006	0.115	0.003	0.955	0.994	0.793–1.245
Germany	0.31	0.111	7.804	0.005	1.363	1.096–1.694
Spain	0.558	0.114	24.113	<.001	1.747	1.398–2.181
Greece	1.007	0.118	73.232	<.001	2.737	2.173–3.449
Ireland	0.97	0.112	74.471	<.001	2.638	2.117–3.290
Education (ref = None/basic)						
Secondary	0.097	0.088	1.209	0.272	1.102	0.927–1.309
University	0.373	0.091	16.73	<.001	1.452	1.214–1.737
Economic Situation (ref = Very bad)						
Bad	0.925	0.143	41.544	<.001	2.522	1.904–3.340
Fair	1.544	0.133	133.848	<.001	4.683	3.607–6.086
Good	2.046	0.139	216.231	<.001	7.737	5.894–10.166
Very good	3.199	0.184	302.67	<.001	24.508	17.099–35.128
Employment Status (ref = Inactive)	0.457	0.08	32.88	<.001	1.579	1.351–1.848
No religiosity (ref = Yes)	0.357	0.069	26.549	<.001	1.429	1.247–1.637
Cohabitation (ref = No)	-0.197	0.08	6.059	0.014	0.821	0.703–0.961
**Threshold**						
Cutpoint 1 (SRH ≤ 1 vs ≥ 2)	-3.499	0.246	202.439	<.001		
Cutpoint 2 (SRH ≤ 2 vs ≥ 3)	-1.174	0.229	26.269	<.001		
Cutpoint 3 (SRH ≤ 3 vs ≥ 4)	0.887	0.229	14.953	<.001		
Cutpoint 4 (SRH ≤ 4 vs ≥ 5)	3.28	0.233	197.595	<.001		

Statistic	Value
-2 Log-likelihood (intercept-only)	9516.49
-2 Log-likelihood (final)	8400.67
LR χ^2 (df=21)	1115.819 (p<.001)
Pseudo R^2 (Cox & Snell)	0.252
Pseudo R^2 (Nagelkerke)	0.27
Pseudo R^2 (McFadden)	0.108

Notes. Ordered logit for SRH (1=very bad-5=very good), fully adjusted; coefficients are log-odds (b), OR=exp(b), with standardizedβ and reference categories in parentheses. Cutpoints are model thresholds (not covariate effects). N=3,844.
